# Acute Kidney Injury after Heart Valve Surgery in Elderly Patients: any Risk Factors to Modify?

**DOI:** 10.21470/1678-9741-2019-0483

**Published:** 2021

**Authors:** Yolanda Carrascal, Gregorio Laguna, Miriam Blanco, Lucia Pañeda, Bárbara Segura

**Affiliations:** 1 Department of Cardiac Surgery, Hospital Clínico Universitario de Valladolid, Valladolid, Spain.

**Keywords:** Cardiac Surgical Procedures - adverse effects, Cardiopulmonary Bypass, Risk Factors, Anemia, Acute Kidney Injury, Aged

## Abstract

**Introduction:**

Postoperative acute kidney injury contributes to longer hospital stays and increased costs related to cardiac surgery in the elderly. We analyse the influence of the patient’s age on risk factors for acute kidney injury after cardiac valve surgery.

**Methods:**

We evaluated the prevalence and risk factors for acute kidney injury in 939 consecutive patients undergoing valve surgery, between 2013 and 2018.

**Results:**

The prevalence of acute kidney injury was 19.5%. Hypertension (P=0.017); RR (95% CI): 1.74 (1.10-3.48), age ≥70 years (*P*=0.006); RR (95% CI): 1.79 (1.17-2.72), preoperative haematocrit <33% (*P*=0.009); RR (95% CI): 2.04 (1.19-3.48), glomerular filtration rate <60 ml/min/1.73 m^2^ (*P*<0.0001); RR (95%) CI: 2.36 (1.54-3.62) and cardiac catheterization <8 days before surgery (*P*=0.021); RR (95% CI): 2.15 (1.12-4.11) were identified as independent risk factors. In patients older than 70 years, with no kidney disease diagnosed preoperatively, glomerular filtration rate <70 ml/min/1.73 m^2^, male gender, cardiopulmonary bypass time, preoperative haematocrit <36% and preoperative therapy with angiotensin-converting enzyme inhibitors were risk factors for acute kidney injury after valve surgery.

**Conclusions:**

In elderly patients, postoperative acute kidney injury develops with higher values of preoperative glomerular filtration rate than those observed in a younger population. Preoperative correction of anaemia, discontinuation of angiotensin-converting enzyme inhibitors and surgical techniques reducing cardiopulmonary bypass time would be considered to reduce the prevalence of renal failure.

**Table:** 

Abbreviations, acronyms & symbols				
**ACE**	**= Angiotensin-converting enzyme**		**HUGE**	**= Haematocrit, blood urea and gender**
**ACEF**	**= Age, creatinine and ejection fraction**		**ICU**	**= Intensive care unit**
**AKI**	**= Acute kidney injury**		**PVC**	**= Polyvinyl chloride**
**AKIN**	**= Acute Kidney Injury Network**		**RIFLE**	**= Risk, Injury, Failure, Loss, End-stage kidney disease**
**CABG**	**= Coronary artery bypass grafting**		**ROC**	**= Receiver operating characteristic**
**COPD**	**= Chronic obstructive pulmonary disease**		**RRT**	**= Renal replacement therapy**
**CPB**	**= Cardiopulmonary bypass**		**SPSS**	**= Statistical Package for the Social Sciences**
**eGFR**	**= Estimated glomerular filtration rate**		**TAVI**	**= Transcatheter aortic valve implantation**
**GFR**	**= Glomerular filtration rate**		**WHO**	**= World Health Organization**

## INTRODUCTION

Postoperative acute kidney injury (AKI) is a known complication after cardiac surgery under cardiopulmonary bypass (CPB). The loss of pulsatility flow, kidney injury related to ischemia and reperfusion, haemolysis, inflammatory response activation, vascular redistribution and embolic phenomena are some of the factors involved in its appearance. Risk factors related to the patient (such as age and preoperative renal failure) increase the probability of the development of AKI. The prevalence of AKI after CPB ranges between 28 and 94% of patients. Between 1 and 10% of patients with AKI require renal replacement therapy (RRT)^[1,2]^. The diagnosis of AKI is related with increased morbidity and mortality in the short, medium and long term, longer hospital stay and increased costs and rates of hospital readmissions (even when serum creatinine levels returns to the normal range before discharge)^[1,2]^.

Population ageing has led to an increasing number of valve surgery procedures among elderly patients. In this group, the morbidity due to postoperative AKI contributes to longer hospital stays and increased costs related to surgery^[1,3]^. We aim to analyse risk factors and predictors of postoperative AKI among patients undergoing cardiac valve surgery at our centre. We also intend to identify the most prevalent risk factors among elderly subjects and the preventive measures accessible in this age group.

## METHODS

The study protocol (identification number PI 17-649, CINV 17-03) received full approval from the local Institutional Research Review Committee and the Clinical Research Ethics Committee. It complies with the ethical guidelines of the Declaration of Helsinki. Informed consent was obtained from each patient. We analysed the influence of different independent risk factors (prospectively collected between 2013 and 2018) on postoperative AKI in a group of 939 consecutive patients undergoing isolated heart valve surgery under CPB. No patient was excluded or lost during recruitment. The analysed variables were prospectively collected from our clinical database.

### Definitions

Patients who met any of the RIFLE classification criteria^[4]^ were diagnosed as AKI. Mortality was defined as 30-day in-hospital mortality after surgery. Urgent surgery was performed during the first 24 hours after surgical indication. Preoperative renal failure was considered according to the EuroSCORE II criteria^[5]^. The diagnosis of chronic obstructive pulmonary disease (COPD) required bronchodilator treatment. The preoperative values of creatinine corresponded to those obtained 48 hours before surgery, except in cases of urgency in which creatinine levels considered correspond to the day of surgery. The estimated glomerular filtration rate (eGFR) was obtained by applying the Cockcroft-Gault formula. Preoperative and intraoperative variables considered are detailed in Table 1.

**Table 1:** Preoperative characteristics of patients under heart valve surgery.

Risk factor				N=939 (%)
**Qualitative**	**Preoperative**	Female gender		407 (43.3)
		Smoking		257 (27.4)
		Hypertension		605 (64.4)
		Peripheral vascular disease		78 (8.3)
		Diabetes mellitus		154 (16.4)
		Dyslipidaemia		442 (47.1)
		Renal impairment* (according EuroSCORE II criteria)	On dialysis (regardless serum creatinine)	1 (0.001)
			Moderately impaired (CC=50-85 ml/min)	442 (47.1)
			Severely impaired (CC <50 ml/min)	93 (9.9)
		Chronic pulmonary disease		63 (6.7)
		Stroke		54 (5.8)
		NYHA III-IV functional class		397 (42.2)
		Sinus rhythm		755 (80.5)
		Severe left ventricular dysfunction (LVEF <30%)		13 (1.3)
		Urgent surgery		54 (5.8)
		Redo surgery		123 (13.1)
		Preoperative haematocrit >33%		824 (87.8)
		eGFR (ml/min/1.73 m^2^)	<70	304 (32.4)
			<60	179 (19.2)
			<50	93 (9.9)
		Valve surgery	Aortic	481 (51.2)
			Mitral	202 (21.5)
			Tricuspid	12 (1.2)
		Preoperative treatment	Beta-blocker	352 (37.5)
			Calcium antagonist	83 (8.8)
			Diuretics	498 (53)
			ACEI	443 (47.2)
**Quantitative**	**Preoperative**			**Media (DS)**
		Age (years)		68.28 (10.64)
		BMI		27.4 (4.4)
		Haemoglobin (g/dl)		12.7 (4.8)
		Haematocrit (%)		38.7 (6.1)
		Creatinine (mg/dl)		1.09 (0.7)
		eGRF (ml/min/1.73 m^2^)		83.6 (38.6)
	**Perioperative**	CPB time (min)		108.8 (47.3)
		Aortic cross-clamp time (min)		83.1 (51.8)
		EuroSCORE logistic		8.55 (8.66)
		Coronary angiography/surgery delay (days)		114.6 (109.3)
		EuroSCORE II		4.67 (6.52)

* According EuroSCORE II criteria. ACEI=angiotensin-converting enzyme inhibitors; BMI=body mass index; CC=creatinine clearance; CPB=cardiopulmonary bypass; eGFR=estimated glomerular filtration rate; RRT=renal replacement therapy

The patients were premedicated with oral zopiclone and subcutaneous morphine. Monitoring was performed by electrocardiogram, pulse oximetry, oesophageal temperature, central venous pressure, blood pressure, urine output (bladder catheter) and pulmonary artery pressure (Swan-Ganz catheter). Fentanyl (5-10 mg/kg iv), etomidate (0.25 mg/kg iv) and rocuronium (1 mg/kg iv) were used in anaesthetic induction. Maintenance was performed with propofol (2-4 mg/kg/h iv), remifentanil (0.15-0.5 mg/kg/min), inspired sevoflurane 0.5-2% and rocuronium (bolus of 0.3 mg/kg).

All procedures were performed under CPB and moderate hypothermia (35ºC) and alpha-stat acid-base strategy management. Aortic and cavo-atrial cannulations were used in aortic valve surgery patients and bicaval cannulation in patients undergoing concomitant mitral and tricuspid valve procedures. After aortic clamping, a mean dose of 750 ml of blood cardioplegic solution (4ºC) (anterograde and retrograde) was administered, repeating infusion (300 ml) every 15-20 minutes. Cardioplegic reperfusion solution (37ºC) was administered before aortic unclamping. CPB was established with a roller pump, a highly haemocompatible polyvinyl chloride (PVC) perfusion tubing coated with phosphorylcholine molecule, and an Inspire 6 (Livanova PLC-London) oxygenator. Mean flow values during CPB were 4.35±1.16 L/min (2.5-2.8 L/min/m^2^). Systemic perfusion pressure was maintained at 60±5 mmHg.

### Statistical analysis

Statistical analysis was performed using SPSS software version 22.0. Quantitative variables were expressed as mean ± standard deviation or as median, for asymmetric distributions. The Kolmogorov-Smirnov test was used to determine the normal distribution of each variable. Qualitative variables were expressed as absolute values and percentages. Association between variables was identified by the χ^2^ or Fisher’s exact test when qualitative and by Student’s t-test or Mann Whitney U-test when quantitative. Association between risk factors and postoperative AKI in the univariate analysis (*P*<0.2) was entered stepwise into multivariate logistic models. A P value <0.05 was considered significant.

## RESULTS

The prevalence of postoperative AKI was 19.5%. The overall mortality was 6%, rising to 12% in AKI patients. Only 7 patients required RRT (0.7%). Mortality in this group rises to 57%. Median intensive care unit (ICU) stay in AKI patients was 5 days (1-44) and 3 days (1-51) in the non-AKI group. Mean hospital stay was 13 days (1-107) in AKI patients and 7 days (1-12) in the non-AKI group. Among the patients, 67.9% (637) were older than 65 years, 54.1% (508) older than 70 years and 13.6% (128) older than 80 years. The prevalence of preoperative renal failure was 6.3% considering the total group, and 8.2%, 9.3% and 12.5% among patients older than 65, 70 and 80 years, respectively. Preoperative eGFR was lower than 60 ml/min/1.73 m^2^ in 19% of the patients analysed. In patients older than 65 years, 25% presented an eGFR <60 ml/min/1.73 m^2^. This percentage increased to 27 % and 36% in patients older than 70 and 80 years, respectively.

**Fig. 1:**
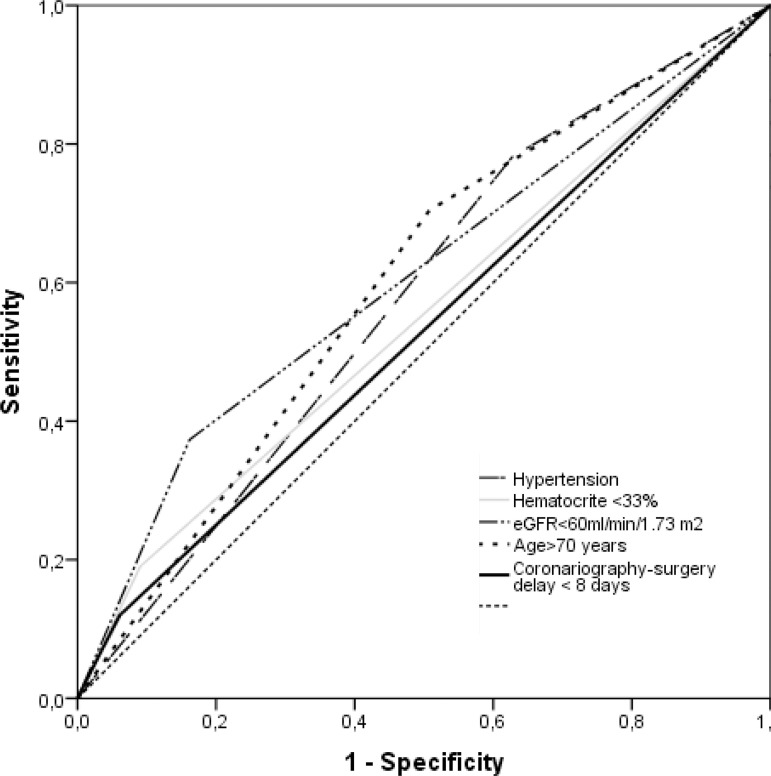
Changes of white blood cells (WBC) and C-reactive protein (CRP) levels in two groups

Preoperative variables analysed in AKI and non-AKI patients are detailed in Table 2. In the multivariate analysis, age over 70 years, preoperative eGFR <60 ml/min/1.73 m^2^, preoperative haematocrit <33%, hypertension and <8 days between coronary angiography and surgery were independent risk factors for the occurrence of postoperative AKI (Table 3). These variables, when combined, provided a model that accurately predicts postoperative AKI with a ROC curve of 0.783±0.036 (95% CI 0.713-0.854, *P*<0.001) (Table 4). (Figure 1) Among the patients older than 70 years without a diagnosis of preoperative renal failure, therapy with angiotensin-converting enzyme (ACE) inhibitors, CPB duration, male gender and preoperative haematocrit <36% were significant risk factors for AKI (Table 5). Unlike in younger adults, preoperative eGFR <70 ml/min /1.73 m^2^ is a significant predictor for postoperative AKI among elderly patients. When considering patients over 80 years, eGFR <70 ml/min/1.73 m^2^ was a significant predictor of postoperative acute renal failure as well as male gender and age. However, we did not observe the influence of preoperative haematocrit, intravenous contrast or ACE inhibitors therapy before surgery in postoperative AKI.

**Table 2:** Risk factors in patients with and without AKI after heart valve surgery.

Variable				Non-AKIN=756 (%)	AKIN=183 (%)	*P*
**Qualitative**	**Preoperative**	Female gender		335 (44.4)	71 (38.8)	0.17
		Smoking		217 (28.7)	40 (21.9)	0.062
		Hypertension		466 (61.6)	139 (76)	<0.0001
		Peripheral vascular disease		60 (7.9)	18 (9.8)	0.40
		Diabetes mellitus		118 (15.6)	36 (19.7)	0.18
		Dyslipidaemia		359 (47.5)	83 (45.4)	0.60
		Moderate-severe renal impairment*		227 (30)	23 (74.3)	<0.001
		Chronic pulmonary disease		47 (6.2)	16 (8.7)	0.22
		Stroke		43 (5.7)	11 (6)	0.86
		NYHA III-IV functional class		312 (41.3)	85 (46.5)	0.23
		Sinus rhythm		605 (80)	150 (82)	0.55
		Severe left ventricular dysfunction (LVEF <30%)		12 (1.6)	0	0.14
		Urgent surgery		38 (5)	16 (8.7)	0.053
		Redo surgery		98 (12.9)	25 (13.6)	0.12
		Preoperative haematocrit >33%		72 (10.3)	34 (20.5)	<0.0001
		Preoperativetreatment	Beta-blocker	288 (38.1)	64 (35)	0.43
			Calcium antagonist	64 (8.5)	19 (10.4)	0.41
			Diuretics	378 (50)	120 (65.6)	<0.0001
			ACEI	342 (45.2)	101 (55.2)	0.016
			Coronary angiography/surgery delay <8 days	42 (6.8)	18 (11.3)	0.058
		eGRF (ml/min/1.73 m^2^)	<70	201 (26.6)	103 (56.3)	<0.0001
			<60	115 (15.3)	64 (35.8)	<0.0001
			<50	55 (7.3)	38 (21.1)	<0.0001
	**Perioperative**	Valve surgery	Aortic	393 (41.8)	88 (9.3)	0.43
			Mitral	165 (15.5)	37 (3.9)	0.73
			Tricuspid	10 (1.06)	2 (0.2)	0.89
			Polivalvular	189 (20.1)	55 (5.8)	0.17
	**Postoperative**	RRT		0 (0)	7 (3.8)	<0.0001
		Mortality		34 (4.5)	22 (12)	< 0.0001
**Quantitative**	**Preoperative**	Age (years)		67.3 (10.8)	72.1 (8.5)	<0.0001
		BMI		27.4 (4.4)	27.5 (4.4)	0.76
		Haemoglobin (g/dl)		12.8 (4.6)	12.2 (5.7)	0.15
		Haematocrit (%)		39.1 (5.9)	36.6 (6.8)	<0.0001
		Creatinine (mg/dl)		1.06 (0.7)	1.2 (0.4)	0.008
		GFR (ml/min/1.73 m^2^)		86.06 (31.2)	73.4 (59.3)	<0.0001
		CPB time (min)		106.8 (44)	117.3 (58.3)	0.007
		Aortic cross-clamp time (min)		82.1 (53.1)	87.6 (45.6)	0.19
		EuroSCORE logistic		8.3 (8,7)	9.5 (8.4)	0.071
		EuroSCORE II		4.4 (6.5)	5.5 (6.2)	0.042
		Coronary angiography/surgery delay (days)		112.1 (108.5)	124 (111.9)	0.21
	**Postoperative**	ICU stay (days)		3.3 (3.5)	6.3 (5.7)	<0.0001

* According EuroSCORE II criteria. ACEI=angiotensin-converting enzyme inhibitors; BMI=body mass index; CPB=cardiopulmonary bypass; eGFR=estimated glomerular filtration rate; ICU=intensive care unit; RRT=renal replacement therapy

**Table 3:** Risk factors for AKI after cardiac surgery in all analysed patients (multivariate analysis).

Risk factor	RR	95% CI	*p*
Hypertension	1.74	1.10-2.76	0.017
Age ≥70 years	1.79	1.17-2.72	0.006
Coronary angiography/surgery delay <8 days	2.15	1.12-4.11	0.021
eGFR <60 ml/min/1.73 m^2^	2.36	1.54-3.62	<0.0001
Preoperative haematocrit <33%	2.04	1.19-3.48	0.009

eGFR=estimated glomerular filtration rate

**Table 4:** Prediction model of acute kidney injury using the risk factors resulted from the multiple logistic regression analysis.

Risk factor	ROC curve*	SE	95% CI	*p*
Hypertension	0.577	0.026	0.527-0.627	0.004
Age ≥70 years	0.598	0.026	0.548-0.649	<0.0001
Coronary angiography/surgery delay <8 days	0.529	0.028	0.475-0.584	0.277
eGFR <60 ml/min/1.73 m^2^	0.606	0.026	0.551-0.661	<0.0001
Preoperative haematocrit <33%	0.550	0.028	0.495-0605	0.066

* Hosmer-Lemeshow (H-L) value=0.720 SE=Standard error. eGFR=estimated glomerular filtration rate

**Table 5:** Risk factors for AKI after heart valve surgery in patients older than 70 and 80 years (multivariate analysis).

Risk factor			
Patients older than 70 years	RR	95% CI	*p*
eGFR <70 ml/min/1.73 m^2^	3.34	1.95-5.72	< 0.0001
Preoperative haematocrit <36%	2.19	1.24-3.86	0.0067
Male gender	1.73	1.006-2.98	0.047
Preoperative ACEI therapy	1.75	1.002-3.05	0.049
CPB time (min)	1.0061	1.0006-1.0116	0.0287
Coronary angiography/surgery delay <8 days	2.30	0.90-5.89	0.0512
Patients older than 80 years	RR	95% CI	*P*
eGFR <70 ml/min/1.73 m^2^	2.99	1.14-7.83	0.0064
Male gender	3.08	1.11-8.51	0.029
Age	1.47	1.11-1.94	0.0064
Preoperative haematocrit <36%	2.65	0.99-7.05	0.0509

ACEI=angiotensin-converting enzyme inhibitors; CPB=cardiopulmonary bypass; eGFR=estimated glomerular filtration rate

In all study population, preoperative haematocrit <33% (*P*=0.0224); RR 5.91 (95% CI 1.28-27.16) and urgent surgery (*P*=0.0203); RR 9.53 (95% CI 1.41-63.99) were identified as incremental risk factors for RRT. In patients older than 70 years, lower preoperative haematocrit was the only significant predictor of RRT (*P*=0.020); RR 0.80 (95% CI 0.66-0.86).

## DISCUSSION

Preoperative identification of AKI risk factors in elderly patients after cardiac surgery would allow the opportunity to correct the surgical risk quantification as well as the implementation of preventive measures. In addition, it should help the Heart Team to choose the most appropriate therapeutic strategy. The interest of preoperative identification of AKI risk factors increases when age-related physiological derangement associated other co-morbidities. Up to now, the validated risk scores referred only to the prediction of postoperative RRT^[6]^, although less severe kidney injury is known to be associated with higher long-term mortality^[7-9]^. Most studies are mainly focused on patients undergoing CABG^[1,2]^ or mixed different diseases^[7]^ and few evaluated the risk of AKI after valve surgery procedures^[10,11]^, one of the most frequent among elderly patients.

According to several studies, the prevalence of postoperative AKI ranges between 28 and 94% and between 1 and 10% of patients require RRT^[1-3]^. Variability in the prevalence of AKI may be due to different evaluation methods: RIFLE^[6]^ (Risk, Injury, Failure, Loss, End-stage kidney disease) KDIGO^[12]^ (Kidney Disease: Improving Global Outcomes) AKIN^[13]^(Acute Kidney Injury Network) or ACEF score (Age, Creatinine and Ejection Fraction)^[14]^ are the most frequently used criteria. Among our patients, AKI was diagnosed within the first 7 days after surgery, according to the RIFLE criteria. Quantification of renal function by means of diuresis volume was not considered in our study, as postoperative fluid management and diuretic treatment could affect it^[7]^.

The prevalence of AKI in our population (around 20%) was comparable to other samples, in which postoperative mortality increases 1.5 to 5 times, and up to 10 times when RRT was required^[1,2,15,16]^. The observed mortality increased 2 times in case of AKI and 14 times when RRT was required. The AKI impact on ICU and hospital stays (increased 2 days in the first case and almost 2 times in the second) is similar to that previously published by other groups^[1,2]^. Age over than 70 years was an independent risk factor. In this group, mortality was significantly higher when AKI was present (11.2% *vs*. 6.8%) (*P*=0.025) and reached 75% of patients submitted to RRT. Among octogenarians, the mortality increased 2 times and reached 100% in the RRT group^[10]^.

Variations in normal glomerular filtration rate (GFR) are related to age, sex and body mass index, ranging from 90-140 ml/min/1.73 m^2^ in healthy young adults. Among elderly patients, small increases in serum creatinine correspond to a greater GRF rate disruption than in younger ones^[17]^. The rate of physiological reduction of GRF is 0.7-1 ml/min/1.73 m^2^/year from 40 years old^[18]^ and rises to 1.05 ml/min/1.73 m^2^ yearly in patients older than 70 years old^[19]^. When GFR ranges between 60-89 ml/min/1.73 m^2^ without kidney damage (absence of proteinuria, normal sediment and kidney image), individuals are classified as decreased GFR (a common situation in elderly), and no specific treatment is advised^[12]^. In the analysed population, relationship between eGFR and age was similar to that described in EUROCAP study (2007), in which an evaluation by general practitioners in a Spanish population showed that 25% of individuals over 65 years old had a GFR <90 ml/min/1.73 m^2^ ^[20]^.

Although the reduction of preoperative eGFR has been identified as a risk factor for postoperative AKI, the cut-off point proposed for patients aged over 70 years is 10 ml/min/1.73 m^2^, higher than the cut-off point for the total population and younger adults^[8]^. Therefore, we can suppose that subclinical forms of kidney dysfunction act as AKI predictors after valve surgery in the elderly.

Few methods for measuring GFR are properly adjusted to be representative over 70 years of age. The eGFR calculation according to Cockcroft-Gault equation over-estimates renal failure in this group, due to the high weight of age. In addition, the Cockcroft-Gault equation has not been reformulated for serum creatinine values obtained by the current procedures (which are 10 to 20% higher than the classic one). All of this leads to eGFR overestimation. Some authors advise the use of more sensitive and specific formulas for AKI screening in the elderly, as haematocrit, blood urea and gender (HUGE) formula, which considers the influence of haematocrit, urea and gender as risk factors^[21]^. According to these findings, we found that, in patients older than 70 years, gender and preoperative anaemia have a greater impact on postoperative AKI manifestation (significantly more often when preoperative haematocrit <36% *vs*. 33% in the general population). In conclusion, eGFR is an adequate predictor of AKI after valve surgery in our elderly population, even admitting that Cockcroft-Gault equation could not be the most appropriate method for its quantification. Probably the HUGE formula will be more sensitive and specific in these patients.

Recent contrast administration and AKI are significantly associated^[22]^. In our elderly patients, contrast-induced nephropathy is enhanced by the frequent association of preoperative anaemia and ACE-inhibitors therapy, as well as CPB time. In patients with preoperative GFR <70 ml/min/1.73 m^2^ (classified as decreased GFR), an interval of at least 8 days should be recommended between the intravenous contrast administration and surgery. Despite the proposed nephroprotective effect of ACE inhibitors, their association with dehydration and diuretic treatment are a common trigger of AKI among the elderly. AKI secondary to overdose of ACE inhibitors is more common after therapy with longer-acting ACE inhibitors, such as enalapril^[23]^.

Of the patients older than 70 years analysed, 76% had hypertension and 56.5% were preoperatively treated with ACE inhibitors. Among elderly people on ACE inhibitors therapy, the eGFR in non-AKI group was 78.15±25.7 ml/min/1.73 m^2^ *versus* 64.16±22.97 ml/min/1.73 m^2^ in AKI group (*P*<0.0001). AKI prevalence was 38.3% when eGFR <75 ml/min/1.73 m^2^, 41.7% when eGFR <70 ml/min/1.73 m^2^, 42.7% if it was <60 ml/min/1.73 m^2^ and 46.7% if eGFR is <50 ml/min/1.73 m^2^. Significant differences in AKI prevalence were observed among patients under ACE inhibitors therapy, only when compared to normal eGRF patients (90-140 ml/min/1.73 m^2^). Therefore, it seems that the influence of ACE inhibitors on AKI after valve surgery was not associated with the preoperative decrease in GRF. Probably it is due to other mechanisms, apart from accumulation or overdosing (which some authors have identified when the GFR is below 30-40 ml/min/1.73 m^2^)^[16,23]^. In the population older than 80 years analysed, preoperative ACE inhibitors therapy was not identified as a risk factor for AKI, probably due to the low number of patients in the sample. The same applies to CPB time, which is significantly shorter in this age group (88.8±42.2 *vs*. 102.7±46.2 min) (*P*<0.0001). Isolated aortic valve procedures were more prevalent in octogenarians (71.8%), whilst among younger patients 27% underwent multivalvular surgery and 47% isolated aortic valve procedures. According to our data, in more than 73% of patients older than 70 years preoperative eGRF is <90 ml/min/1.73 m^2^. We can conclude that it would be advisable to discontinue ACE inhibitor treatment before heart valve surgery, regardless of eGFR value.

The WHO (World Health Organization) fixed the lower limit of Hb at 13 g/dL in men and 12 g/dl in women to diagnose anaemia. Preoperative anaemia is a risk factor for AKI in all patients but is more powerful in those older than 70 years. During surgery, kidney injury occurs by association of hypoxia (secondary to haemodilution), release of iron and proinflammatory agents. Transfusion requirements and free plasma haemoglobin cause a decrease in haptoglobin, making unable the free radicals neutralization^[14]^. Consequently, in all patients, but especially in those older than 70 years, preoperative anaemia correction techniques (B12 or iron supplementation, growth factors etc.) are mandatory even in mild cases in which red blood cells transfusion is not recommended. Complementary techniques, such as autotransfusion and mini-CPB circuits, might also reduce haemodilution in this case. The haematocrit values during CPB should be greater than 20%, avoiding fluid overload. Intravenous mannitol infusion or intraoperative ultrafiltration can prevent this complication^[14]^.

CPB duration is an independent risk factor for AKI in elderly patients. Interventions that reduce or avoid its use should decrease the prevalence of renal damage. Although the techniques for percutaneous mitral valve replacement are not yet perfectly developed, there are two alternatives to conventional treatment of aortic valve disease (one of the most prevalent diseases in the elderly). Transcatheter aortic valve implantation (TAVI) seems to be associated with lower AKI (between 8.3 and 26% *vs*. 43.7%) and RRT risk (2-3% *vs*. 7.6%) regarding to surgery and reduce the need for transfusion^[24]^. However, some authors have identified a risk of 57% or even higher when transapical technique is used. In addition, the administration of more than 99 ml of intravenous contrast during procedure significantly increases the risk of AKI^[11]^. Although the results of mortality and AKI prevalence are favourable in patients at high surgical risk, in low-risk patients (EuroSCORE II: 4%) it is associated with increased mortality in 3 years and increased perioperative risk of stroke, need for permanent pacemaker, paravalvular aortic regurgitation, vascular injury and cardiac tamponade^[25]^. Therefore, its use would not be recommended, even in the elderly. In this case, sutureless aortic valve replacement could be an alternative therapy. Its use is associated to a significant reduction on CPB time, less surgical morbidity (due to associated mini-incision techniques), bleeding reduction, and decreased AKI prevalence (estimated at about 3% of cases)^[26]^. Particularly in older or oldest-old patients, sutureless valve would avoid problems associated with TAVI implantation, which contributed to a significant decrease in medium-term survival.

### Limitations

Although this is a prospective, consecutive and observational study of a homogeneous population, Cockcroft-Gault equation may not be the most appropriate way to measure eGFR among elderly population, as age is over-considered in its calculation.

Although the kidney profile is very similar between the population analysed and the Spanish general population, the results could not be extrapolated outside our borders.

## CONCLUSION

In elderly patients, postoperative AKI after CPB develops with higher preoperative GFR values than those observed in a younger population. Preoperative correction of anaemia, discontinuation of ACE inhibitors and surgical techniques reducing CPB time would contribute significantly to decreased postoperative AKI prevalence.

In this group of patients, the individual patient co-morbidities, estimated surgical risk and AKI risk factors should determine surgical decision-making and adequacy of new therapies that could prevent or reduce the CPB use.

**Table:** 

Authors' roles & responsibilities	
YC	Substantial contributions to the conception or design of the work; or the acquisition, analysis or interpretation of data for the work; drafting the work or revising it critically for important intellectual content; final approval of the version to be published
GL	Drafting the work or revising it critically for important intellectual content;; final approval of the version to be published
MB	Substantial contributions to the conception or design of the work; or the acquisition, analysis or interpretation of data for the work; final approval of the version to be published
LP	Substantial contributions to the conception or design of the work; or the acquisition, analysis or interpretation of data for the work; final approval of the version to be published
BS	Substantial contributions to the conception or design of the work; or the acquisition, analysis or interpretation of data for the work; final approval of the version to be published
